# Optimization of Tomato Shoot Architecture by Combined Mutations in the Floral Activators *FUL2/MBP20* and the Repressor *SP*

**DOI:** 10.3390/ijms26031161

**Published:** 2025-01-29

**Authors:** Xiaobing Jiang, María Jesús López-Martín, Concepción Gómez-Mena, Cristina Ferrándiz, Marian Bemer

**Affiliations:** 1 Shenzhen Branch, Guangdong Laboratory of Lingnan Modern Agriculture, Key Laboratory of Synthetic Biology, Ministry of Agriculture and Rural Affairs, Agricultural Genomic Institute at Shenzhen, Chinese Academy of Agricultural Sciences, Shenzhen 518120, China; xiaobing.jiang@wur.nl; 2 Laboratory of Molecular Biology, Wageningen University & Research, Droevendaalsesteeg 1, 6708 PB Wageningen, The Netherlands; 3 Business Unit Bioscience, Wageningen University & Research, Droevendaalsesteeg 1, 6708 PB Wageningen, The Netherlands; 4 Instituto de Biología Molecular y Celular de Plantas, Consejo Superior de Investigaciones Científicas-Universidad Politécnica de Valencia, 46022 Valencia, Spain; mjlopez@ibmcp.upv.es (M.J.L.-M.); cgomezm@ibmcp.upv.es (C.G.-M.); cferrandiz@ibmcp.upv.es (C.F.); 5 Biosystematics Group, Wageningen University & Research, Droevendaalsesteeg 1, 6708 PB Wageningen, The Netherlands

**Keywords:** shoot architecture, flowering, sympodial growth, tomato

## Abstract

Shoot determinacy is a key trait affecting productivity in tomato, quantitatively governed by genes within the flowering pathway. Achieving an optimal balance of flowering signals is essential for shaping plant architecture and maximizing yield potential. However, the genetic resources and allelic diversity available for fine-tuning this balance remain limited. In this work, we demonstrate the potential for directly manipulating shoot architecture by simultaneously targeting the flowering activating *FRUITFULL*(*FUL*)-like genes, *FUL2* and *MADS*-*BOX PROTEIN 20* (*MBP20*), and the flowering-repressing gene *SELFPRUNING* (*SP*). Loss of *MBP20* in the *sp* background leads to additional inflorescences, while determinacy is largely maintained. However, additional mutation of *FUL2* results in mainly indeterminate plants, which have faster sympodial cycling, leading to more compact growth and increased flower production. Our results provide a path to quantitative tuning of the flowering signals with a direct impact on shoot architecture and productivity.

## 1. Introduction

Plant architecture plays a crucial role in determining crop productivity, including flower, fruit, and seed production. In crops such as maize, rice, and soybean, architectural traits have been modified to meet agricultural conditions like high-density planting and machine harvesting [1,2,3,4]. In tomato, the indeterminate growth habit exhibits minimal variation among wild species and traditional cultivated varieties [5]. However, the discovery of the *self-pruning* (*sp*) natural variant in 1927 marked a breakthrough, as it transformed the indeterminate shoot architecture into a determinate form [6]. This mutation revolutionized tomato cultivation by enabling a more compact growth habit, which is particularly advantageous for field-grown tomato because it facilitates mechanical harvesting and synchronized ripening. Given the limited natural variation, a deep understanding of the genetic interactions underlying tomato shoot determinacy is desired for broad agricultural practices.

The shoot determinacy in tomato is largely determined by the transition from vegetative to reproductive growth. Tomato has a sympodial shoot architecture, which is distinct from that of the monopodial Arabidopsis. Upon floral transition, the tomato shoot apical meristem (SAM) terminates into an inflorescence and the shoot growth resumes from a sympodial shoot meristem (SYM) in the axil of the youngest leaf primordium [7]. The SYM produces three leaves before terminating in the first flower of the next inflorescence, forming a sympodial shoot. This pattern is continuously repeated, enabling indeterminate plant growth. The regularity of sympodial cycling is maintained by a finely tuned balance between flower-promoting and flower-repressing signals [8]. In *sp* mutants, florigen is no longer counteracted, causing shoots to terminate progressively faster until cycling stops [8,9]. *SP* was identified as the ortholog of the Arabidopsis *TERMINAL FLOWER1* (*TFL1*) gene and encodes a repressor of flowering by competing with the florigen *SINGLE FLOWER TRUSS* (*SFT*, ortholog of *FLOWERING LOCUS T* (*FT*)), specifically in the SYMs [9,10]. In contrast to loss of *SP*, loss of *SFT* arrests sympodial shoot growth and leads to highly vegetative plants with very few flowers [11,12]. The dosage effect on *sp* determinacy can also be modified by epistatic interactions between *SP* and other florigen pathway genes [13]. It is notable that *sft*/+ heterozygosity quantitatively compensates for *sp* determinate growth, producing a semi-determinate shoot developing additional inflorescences [14,15]. Similar but weaker effects result from mutations in *SUPPRESSOR OF SP* (*SSP*, ortholog of *FD*), which forms a multimeric complex with florigen to regulate the expression of floral transition genes [15]. The calibrating function of close homologs of *SP* and *SFT* on plant architecture is highly conserved in many other crops, including soybean [4], sunflower [16],cotton [17], and barley [18]. However, despite the agricultural significance of the flowering balance in tomato shoot architecture, the limited number of genes and alleles available may not provide the optimal tools to achieve maximum productivity given the diversity of tomato growth practices.

Recent research has identified several additional genes that influence the sympodial index and, consequently, plant architecture [19,20]. These genes represent a valuable new source for optimizing the balance of flowering signals to maximize productivity. The *FRUITFULL*(*FUL*)-like genes, for example, are critical regulators of sympodial shoot flowering time [19]. In tomato, *FUL1* plays a relatively minor role and appears to depend on *FUL2* and *MADS-BOX PROTEIN 20* (*MBP20*) for its activation in inflorescence and floral meristems [19]. In contrast, *MBP10*, the fourth tomato *FUL*-like gene, has likely lost its functional relevance. Notably, the loss of *FUL2* and *MBP20* function delays flowering while enhancing inflorescence complexity [19]. It is possible that the activity of SP in the SYM is similar to that of TFL1 in Arabidopsis inflorescence meristems, where TFL1 represses *FUL* and its close homolog *AP1* to repress floral meristem initiation [21]. We hypothesized that the flowering-repressing effect of mutations in the *FUL*-like genes in the sympodial shoot, in combination with their role in inflorescence branching, may render them perfect candidates to counteract *sp* mutations to achieve an optimal balance of flowering signals and subsequent increases in tomato yield. Therefore, we investigated the genetic and phenotypic interactions between *SP* and *FUL2* and *MBP20* and determined whether their mutations could lead to a more optimal balance of flowering signals. We demonstrate that the simultaneous mutation of *SP* and *MBP20*, generated with CRISPR/Cas9, improves tomato shoot architecture and potentially increases fruit productivity.

## 2. Results and Discussion

**Figure 1:**
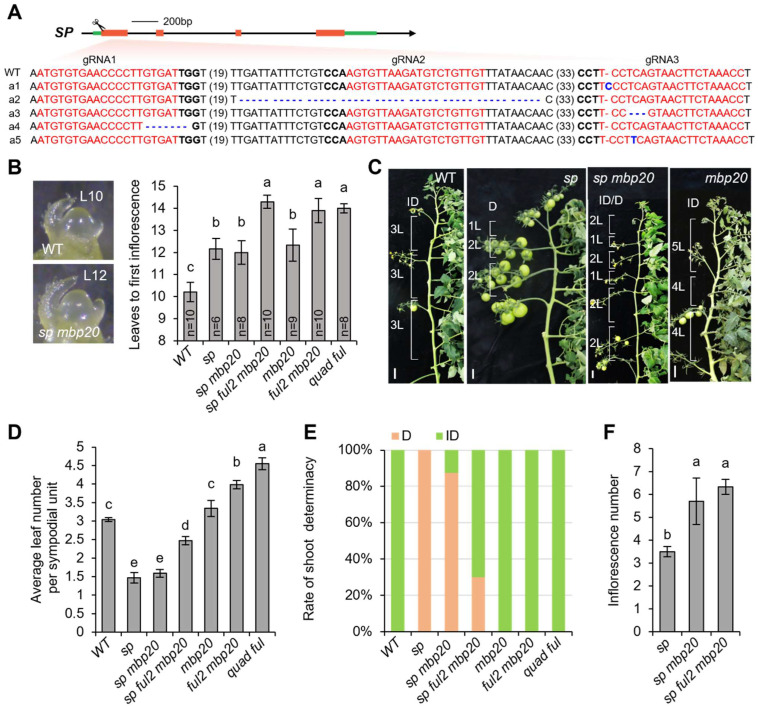
Mutations in *SP* and *FUL2*/*MBP20* can be combined to create variation in shoot architecture compactness. (**A**) Sequences of *SP* alleles (a) obtained with CRISPR/cas9 using three guide RNAs (gRNAs), namely, *sp* (a1, a2), *sp* (a3, a4) *mbp20*, and *sp* (a5) *ful2 mbp20*. The *mbp20* and *ful2 mbp20* lines were previously generated [19]. The gRNA and protospacer-adjacent motif (PAM) sequences are shown in bold red and black, respectively. Deletions and insertions are indicated by blue dashes and blue font, respectively, and the lengths of sequence gaps are indicated in parentheses. (**B**) Quantification of primary shoot flowering time for wild-type (WT) and mutant plants. n: numbers of individual plants measured. *quad ful*: *ful1 ful2 mbp10 mbp20* quadruple mutant (generated in [19]). (**C**) Representative main shoots from all genotypes. Three-month-old plants are shown. L: leaf; D/ID: determinate and indeterminate growth. White bar: 5 cm. (**D**) Average leaf number in sympodial shoots across all genotypes, measured for the first five successive sympodial units. (**E**) Proportion of shoot determinacy of the genotypes. (**F**) Quantification of inflorescence numbers in determinate plants of *sp*, *sp mbp20*, and *sp ful2 mbp20* mutants. In (**B**,**D**,**F**), mean values (±SE) were compared between genotypes using one-way ANOVA followed by a post hoc LSD test. Statistical significance in (**F**) was assessed using the Wilcoxon rank-sum test. Different letters indicate significant differences at the *p* < 0.05 level.

To investigate the genetic interaction between *SP* and *FUL2*/*MBP20*, we used CRISPR/Cas9 to knock out *SP* in the *mbp20* and *ful2 mbp20* mutant backgrounds [19]. The first exon of *SP* was targeted by three guide RNAs (gRNAs) (Figure 1A). The independent first-generation (T0) lines were obtained from stable transformation and analyzed by PCR and sequencing to identify the null mutations. Two presumed null mutant lines were selected, and homozygous T1 progeny plants were used for phenotypic analysis.

Flowering time in the primary shoot was assessed by counting the number of leaves produced before apical doming, which marks the transition to reproductive development (Figure 1B), while sympodial cycling was assessed by counting leaves in the first five sympodial shoots (Figure 1C). Interestingly, we observed delayed primary shoot flowering in *sp* mutants, with the transition occurring after 12 leaves, compared to 10 leaves in the wild-type (WT). This finding contrasts with previous studies, which suggested that the *sp* mutation does not affect primary shoot flowering in tomato cultivar M82 [14,15], likely due to the absence of *SP* expression in the SAM [22]. To validate our observation, we performed an independent trial and observed variable phenotypes, which could be either a small delay or a small acceleration of flowering in the *sp* mutants (Supplementary Figure S1A). Further examination of *SP* expression in the Moneyberg dataset [20] revealed variable yet detectable expression levels in the late-vegetative and transition meristems of the primary shoot (Supplementary Figure S1B). This suggests that *SP* may have a subtle role in regulating vegetative phase transitions in the primary shoot, with its effects being contingent on genetic background and environmental conditions.

When *SP* was mutated in the *mbp20* background, the primary shoot flowering was delayed by 1–2 leaves compared to the WT (Figure 1B), with no significant difference between the single *mbp20* mutant and the *sp mbp20* mutant. This again suggests that *SP* plays at most a minor role in the regulation of primary shoot flowering. The *FUL2* mutation enhanced the phenotype, adding approximately two extra leaves in the *ful2 mbp20*, *sp ful2 mbp20*, and *ful1 ful2 mbp10 mbp20* (*quad ful*) mutants compared to the *mbp20*, *sp mbp20*, and *sp* mutants; however, the *sp* mutation did not exert an additional effect.

In sympodial shoots, WT plants cycled with three leaves per shoot, while *sp* mutants displayed faster cycling until rapid shoot termination, as previously reported [9,15]. Compared to the *sp* mutant, *sp mbp20* had a very comparable sympodial index, with on average 1.5 leaves between two inflorescences (Figure 1C,D, Supplementary Figure S3A). However, a small number of plants displayed indeterminate growth (Figure 1E and Supplementary Figure S4). Moreover, in the determinate plants, on average two extra inflorescences developed in *sp mbp20* double mutants compared to *sp* (Figure 1F). When *FUL2* was additionally mutated, the *sp ful2 mbp20* plants exhibited slower cycling than *sp* and *sp mbp20* mutants, with an average of 2.5 leaves per shoot, although this remained faster than in WT plants (Figure 1C,D, Supplementary Figures S2 and S3B). Remarkably, the indeterminate growth was largely restored in 70% of the *sp ful2 mbp20* plants (Figure 1E, Supplementary Figure S2), validated by an independent trial (Supplementary Figure S4). The reduced sympodial index of *sp ful2 mbp20* mutants will lead to accelerated production, probably without much effect on the production of assimilates. This combination could thus result in enhanced production in the greenhouse.

**Figure 2:**
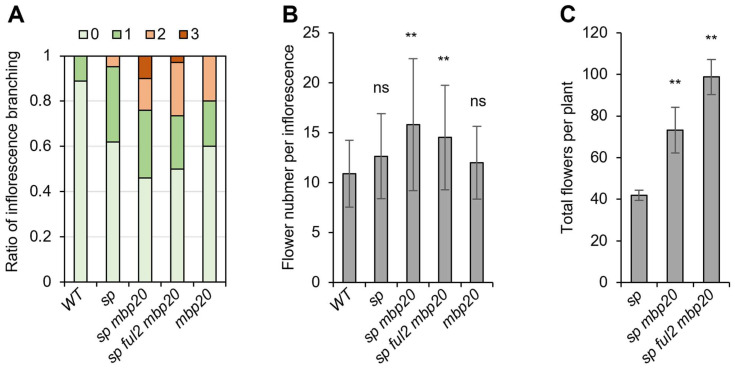
Quantification of flower production. (**A**) Proportion of branched inflorescences per branching category for the indicated genotypes. The numbers (0–3) indicate the number of branching events. (**B**,**C**): Quantification of flower numbers per inflorescence and the total flower number per plant. In (**B**,**C**), mean values (±SD) were analyzed for statistical significance using a *t*-test. Significant differences compared to WT plants (**B**) and *sp* plants (**C**) are represented by asterisks: ** *p* < 0.01. ns: non-significant.

For field production, *sp mbp20* appears to be a suitable combination, not only because it produces two additional inflorescences, but also because *mbp20* mutants display mildly enhanced inflorescence branching without severe effects on flowering time [19]. To investigate whether there is potential for a positive effect on fruit yield, we monitored flower production in these mutants. We performed two trials, the results of which showed that the inflorescence branching trait is highly variable, as also described in refs. [19,20]. However, in all trials, both the number of branching events and the number of flowers per inflorescence were significantly higher than the WT in the *sp mbp20* and *sp ful2 mbp20* mutants (Figure 2A,B and Supplementary Figure S5A,B). Importantly, the determinate *sp mbp20* and *sp ful2 mbp20* mutant plants produce in total more flowers than the *sp* mutants (Figure 2C and Supplementary Figure S5C). Taken together, combining the mutations increases flower production, thus rendering a promising strategy for improving fruit yield.

**Figure 3:**
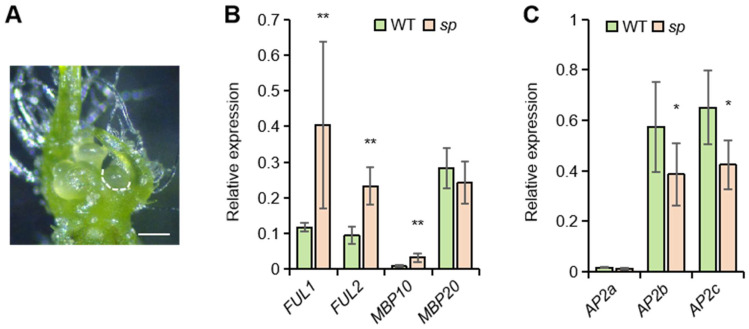
Gene expression analysis in the sympodial vegetative meristems. (**A**) Microdissection of the SYM stage was performed for gene expression analysis. Dashed line represents the boundary of the dissected tissue. White bar: 200 μm. (**B**,**C**) Gene expression of *FUL*-like genes (**B**) and *AP2*-like genes (**C**) in SYM detected by qRT-PCR. The values shown (mean ± SE) are the average of three replicates. Significant differences were calculated using a one-tailed Student’s *t* test (* *p* < 0.05 and ** *p* < 0.01).

Interestingly, our results suggest a dosage-sensitive interaction between *SP* and *FUL2*/*MBP20*, similar to what has been previously found for combinations of *sft* and *sp* alleles [15]. However, where the latter is associated with the florigen/antiflorigen balance, the dosage effect of the tomato *FUL*-like genes may rather be an effect of their redundant activity downstream of SP. Notably, *FUL2* and *MBP20* expressions are upregulated in SYMs of mild *sp* mutants and even more strongly derepressed in *sp* knock-out mutants [13], while *SP* does not appear to be deregulated in *quad ful* mutants, nor in mutants of interactors of FUL2/MBP20 (i.e., *tomato mads 3* (*tm3*) and *sister of tm3* (*stm3*)) [19,20]. We therefore expect that SP is functioning upstream of *FUL2*/*MBP20* to repress their expression in developing SYMs until the floral transition. This is in line with our observed phenotypes, which show that mutations in *FUL2*/*MBP20* can rescue the *sp* knock-out phenotype to some extent. To examine the effect of SP on the tomato *FUL*-like genes in our Moneyberg background, we dissected SYMs from both WT and *sp* mutants to assess gene expression (Figure 3A). Our analysis revealed significantly increased levels of *FUL1*, *FUL2*, and *MBP10* in the *sp* mutant, while *MBP20* expression seemingly remained unchanged (Figure 3B). This finding differs from that of a previous study [13], in which *MBP20* expression was also upregulated in the *sp* mutant. Altogether, the results indicate that SP represses the tomato *FUL*-like genes to delay the floral transition. Additionally, given the link between *AP2*-like gene expression and vegetative fate [20,23,24] and the identification of *AP2*-like genes as downstream targets of FUL2 and MBP20 [19], we assessed *AP2*-like gene expression in *sp* SYMs. The results revealed reduced expression of *AP2b* and *AP2c* in the *sp* mutant (Figure 2C), aligning with the observed faster sympodial flowering phenotype of *sp* mutants. Because the *AP2b* and *AP2c* genes are probably direct downstream targets of the tomato FUL- and SOC1-like proteins [20], it is likely that SP represses the tomato *FUL*-like genes, which in their turn repress *AP2b* and *AP2c*.

The fact that the *sp* knock-out phenotype is not completely rescued by *ful2 mbp20* suggests that there are still other genes downstream of SP that play a role, most likely *FUL1*, *TM3*, or *STM3*, the mutants of which showed mild delays in sympodial shoot flowering [19,20]. It is plausible that the florigen SFT regulates the tomato *FUL*-like genes as well, similar to the mechanism observed in Arabidopsis, where *FUL* is controlled by the FT/TFL1-FD module [21]. The antagonism between SP and SFT appears to be mediated by competition for bZIP transcription factors, which bind to promoters of *FUL*-like genes [15,21]. Thus, our data indicate that *SP* and *FUL2*/*MBP20* are acting in the same pathway, with *FUL2*/*MBP20* being repressed by SP to control the timing of the floral transition of the SYM.

**Figure 4:**
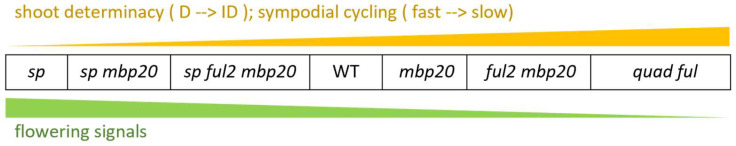
Model of shoot determinacy in relation to *FUL*-like flowering signals. When flowering signals are reduced, sympodial flowering is delayed, resulting in an indeterminate shoot growth habit.

In breeding, dramatic phenotypes are largely avoided due to possible trade-offs. For example, compound inflorescence mutants produce extremely branched inflorescences with hundreds of flowers but set fruit poorly [7], likely due to imbalances in source–sink relationships. However, the mild phenotype in *sp mbp20* mutants resulted in a higher number of flowers per inflorescence and two additional inflorescences per plant, which could be beneficial in agricultural practices. The *sp mbp20* mutants may lead to enhanced yields in field production, while the largely indeterminant *sp ful2 mbp20* mutants with mildly faster sympodial cycling are more suitable for greenhouse cultivation. Notably, while 30% of the *sp ful2 mbp20* plants did terminate primary shoot growth at some point, they resumed growth from the most apical axillary bud and continued to produce sympodial units with an index of approximately 2.5 leaves. Our results suggest that different flowering genes, including *FUL2* and *MBP20*, can be used to genetically tailor antiflorigen effects for the modification of plant architecture (Figure 4). This approach has broader implications for breeding in other Solanaceae crops and beyond, offering opportunities to harness natural variation or engineer new alleles for improved productivity and adaptability.

## 3. Materials and Methods

### 3.1. Plant Materials and Growing Conditions

Seeds of tomato cv. Moneyberg were germinated either on ½ MS for tissue culture transformation or on moistened filter paper for genotyping/phenotyping. Tissue culture transformation was conducted in a growth chamber with 16 h light and 8 h dark at 25 °C. Seedlings from tissue culture and seed germination were transplanted to rockwool and cultivated in a 21 °C growth chamber (16 h light/8 h dark) for several weeks. Finally, plants were transferred to the greenhouse under natural light supplemented with artificial sodium lights.

### 3.2. CRISPR Construct Generation and Stable Tomato Transformation

The *mbp20* and *ful2 mbp20* transgenic CRISPR lines were previously generated [19], and their cotyledons were used together with WT cotyledons for transformation of an *SP* CRISPR/Cas9 construct. The construct was generated using GoldenGate cloning and the MoClo toolkit as described [25]. Briefly, the online program http://www.rgenome.net/cas-designer/ (accessed on 26 August 2020) [26] was used for guide RNA (gRNA) design. Three gRNAs were used to target the first exon of *SP*. Each gRNA was fused to the synthetic U6 promoter as U6p::gRNA, and cut-ligated in Level 1 vectors. Level 1 constructs pICH47732-NOSpro::NPTII::OCST, pICH47742-35S::Cas9::NOST, pICH47751-35S::GFP::ter35S, pICH47761-gRNA1, pICH47772-gRNA2, pICH47781-gRNA3, and the linker pICH41822 were cut-ligated into the level 2 vector pICSL4723 as described. The level 2 plasmid was transformed into *Agrobacterium* strain C58C1. The above constructs were introduced into tomato cv. Moneyberg by *Agrobacterium tumefaciens*-mediated transformation [27]. Homozygous T1 or T2 transgenic plants were used for phenotypic and molecular characterization. All primers are listed in Supplementary Table S1.

### 3.3. Genotyping and Phenotyping

PCRs were conducted directly on sampled leaf tissue using the Phire Plant Direct PCR kit (Thermo Fisher, Landsmeer, the Netherlands), and the mutations were identified using Sanger sequencing of the PCR fragment. Phenotypic analysis of yield component traits was performed for plants of all genotypes grown in the greenhouse. Inflorescence branching was assessed by counting the branching points in at least five inflorescences per plant. The average sympodial leaf index was determined by quantifying the number of leaves in the first five sympodial units. The number of flowers was quantified from the first four inflorescences of indeterminate plants and all inflorescences in determinate plants. Inflorescences were counted only when fully developed.

### 3.4. Meristem Imaging

Plant shoot meristems were manually dissected using a sharp needle and imaged under a stereomicroscope (Stemi 508, Zeiss, Oberkochen, Germany) equipped with an AxioCam IC camera (Zeiss, Oberkochen, Germany).

### 3.5. qRT-PCR Analysis

For qRT-PCR analysis of gene expression, SYMs were harvested at the vegetative stage in triplicate for both wild-type (WT) and mutant plants. For each sample, more than 30 meristems were collected. The replicates were grown sequentially in the greenhouse. The harvested tissue was processed for RNA stabilization using an acetone fixation technique [28]. RNA was extracted using the PicoPure RNA Extraction Kit (Arcturus/Thermofisher, Landsmeer, the Netherlands) and treated with Ambion Turbo DNase (AM1907, Ambion/Thermofisher, Landsmeer, the Netherlands) to remove genomic DNA contamination. cDNA was synthesized with the iScript cDNA Synthesis Kit (Bio-Rad, Mitry-Mory, France). Real-time PCR was performed using the iQ SYBR Green Supermix (Bio-Rad, Mitry-Mory, France) with a standard two-step program of 40 cycles, including annealing/extension at 60 °C. Primer efficiencies were evaluated beforehand, and only primer pairs with comparable efficiencies were used for analysis. The *CAC* gene was employed as a reference for normalization. All primer sequences used in this study are provided in Supplementary Table S1.

### 3.6. Accession Numbers

SP, Solyc06g074350; FUL1, Solyc06g069430; FUL2, Solyc03g114830; MBP10, Solyc02g065730; MBP20, Solyc02g089210; AP2a, Solyc03g044300; AP2b, Solyc02g064960; AP2c, Solyc02g093150.

## Data Availability

Data is contained within the article.

## Supplementary Materials

The following supporting information can be downloaded at: https://www.mdpi.com/article/10.3390/ijms26031161/s1.
