# Supplementary material for: Optimization of Tomato Shoot Architecture by Combined Mutations in the Floral Activators FUL2/MBP20 and the Repressor SP
Source: Int J Mol Sci. 2025 Jan 29;26(3):1161. doi: 10.3390/ijms26031161 (PMC11817714; doi:10.3390/ijms26031161)
Supplement: Supplementary file 1 [file ijms-26-01161-s001.zip › ijms-3412470-supplementary.pdf]

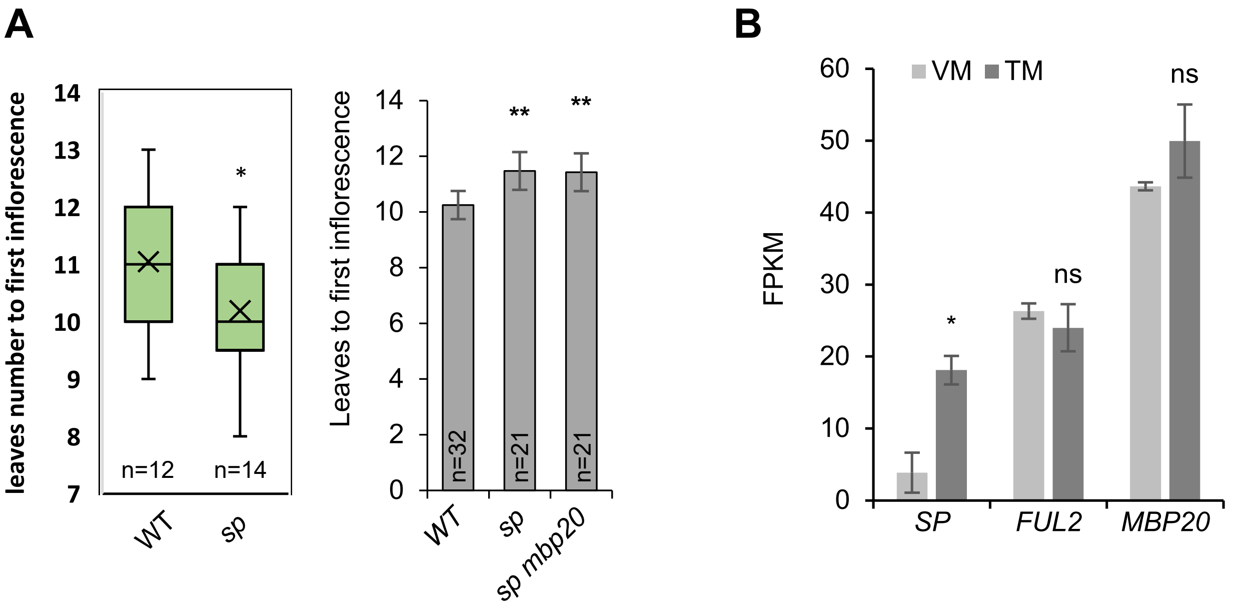

Supplementary Figure 1. *SP* mutation delays primary shoot flowering. (A) Quantification of primary shoot flowering time for wild-type (WT) and mutant plants from two independent trials. n: numbers of individual plants measured. (B) Normalized gene expression (FPKM) of the SP in WT vegetative meristem (VM) and transition meristem (TM). The values shown (mean ± SE) are the average of three replicates. Significant differences were calculated using one-way ANOVA followed by a post hoc LSD test in (A), and one-tailed Student's t test in (B) (*$P < 0.05$, **$P < 0.01$). ns: non-significant.

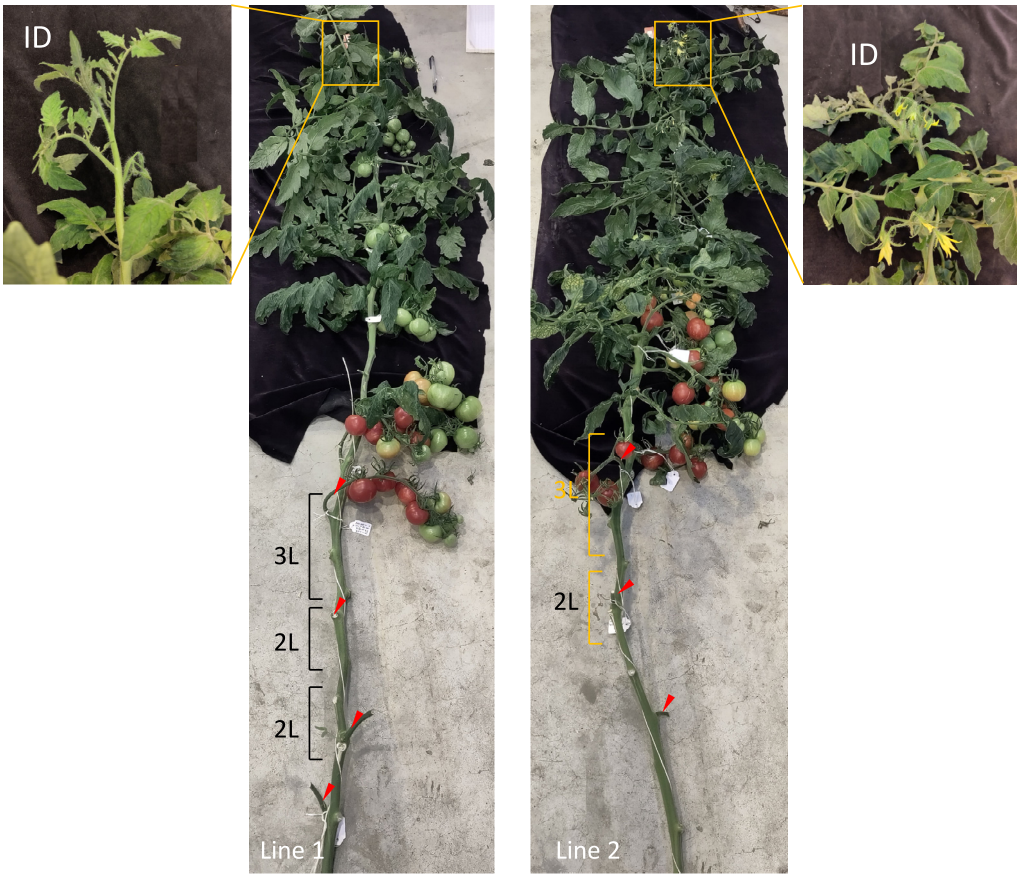

Supplementary Figure 2. Representative main shoots from *sp ful2 mbp20* mutant plants. Three-month-old plants are shown. L: leaf. ID: indeterminate growth. Red triangles indicate removed inflorescences. Leaves are removed to expose the sympodial shoots.

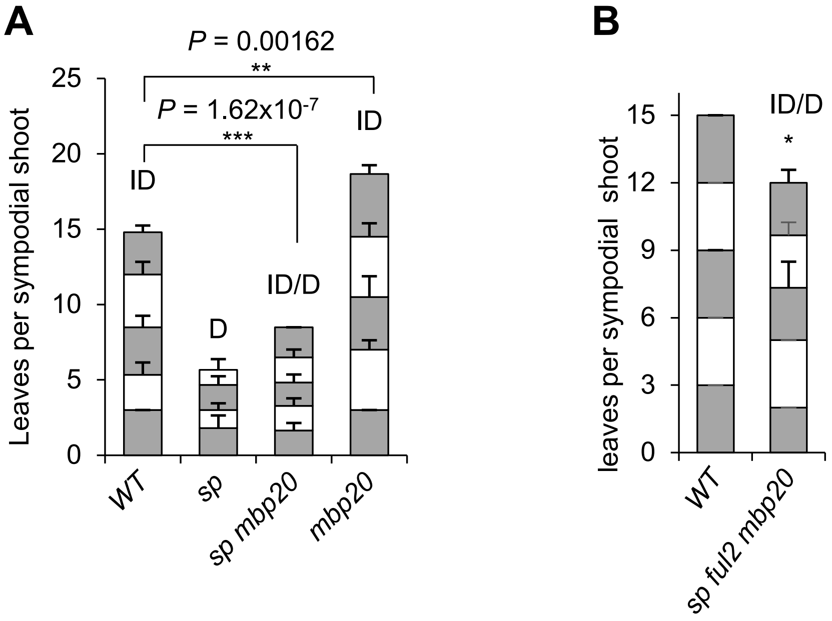

Supplementary Figure 3. ***SP* mutation delays primary shoot flowering.** (**A**) Number of leaves per sympodial unit for the first five units in WT, *sp*, *sp mbp20*, and *mbp20* mutants. (**B**) Number of leaves per sympodial unit for the first five units in WT and *sp ful2 mbp20* mutants. The average of the leaves per unit was used to test the significance. D/ID: determinate and indeterminate growth. Mean values (±SE) were compared between genotypes using one-way ANOVA followed by a post hoc LSD test (**P*<0.05, ***P* < 0.01, and ****P* < 0.001).

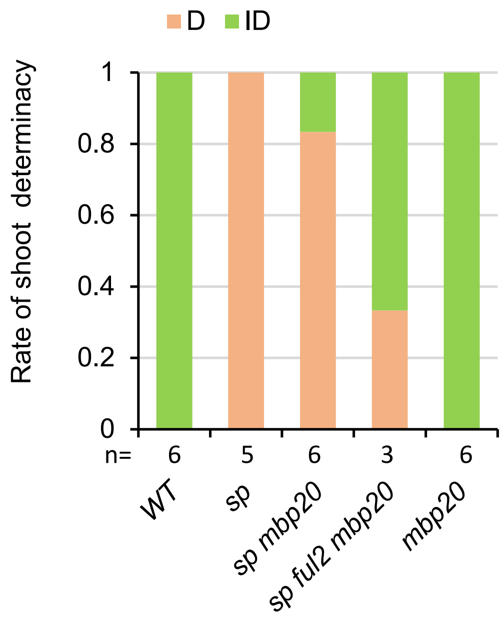

Supplementary Figure 4. Quantification of plant determinacy proportions. D:determinate, ID: indeterminate. n: numbers of individual plants.

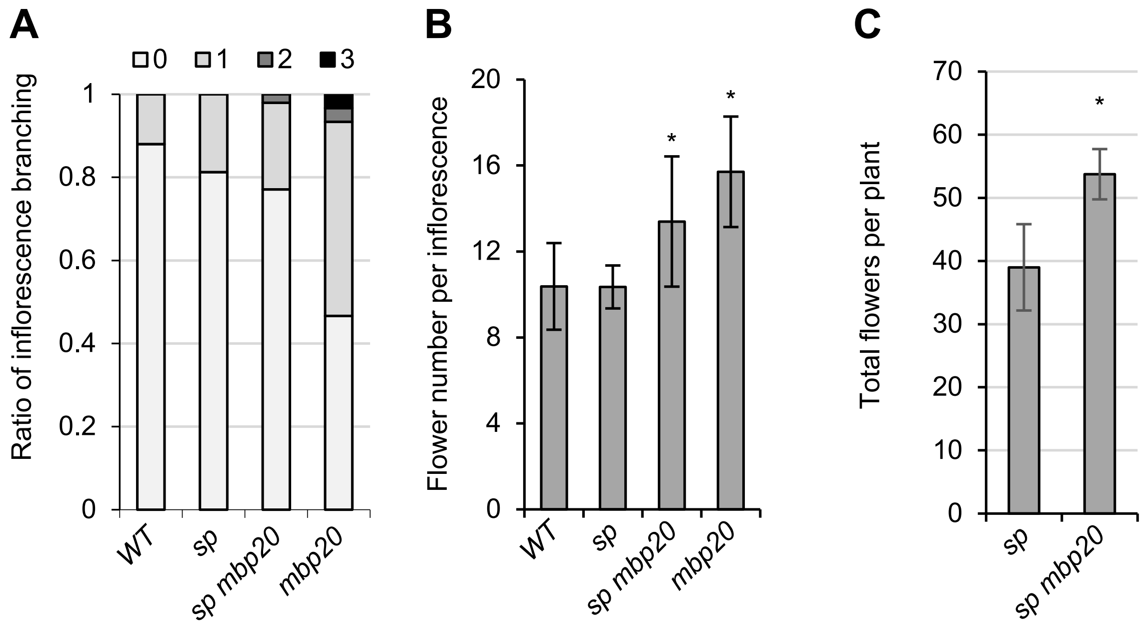

Supplementary Figure 5. Quantification of flower production. (A) Proportion of branched inflorescences per branching category for the indicated genotypes. The numbers (0-3) indicate the number of branching events. (B) and (C) Quantification of flower numbers per inflorescence and total flower number per plant. Mean values (± SE) were analyzed for statistical significance using a t-test. Significant difference compared to *sp* plants is represented by asterisks: $*P < 0.05$.

Supplementary Table 1. Primers used in this study.

Oligo name	Sequence 5' to 3'	purpose
SP-sgRNA-1 F	TGTGGTCTCAATTGATGTGTGAACCCCTTGTGATGTTTTAGAGCTAGAAATAGCAAG	CRISPR
SP-sgRNA-2 F	TGTGGTCTCAATTGACAACAGACATCTTAACACTGTTTTAGAGCTAGAAATAGCAAG	
SP-sgRNA-3 F	TGTGGTCTCAATTGGTTTAGAAGTTACTGAGGAGTTTTAGAGCTAGAAATAGCAAG	
sgRNA universal R	TGTGGTCTCAAGCGTAATGCCAACTTTGTAC	
SP genotyping F	TTGTAGCACGAGAAGAGAATAACCT	genotyping
SP genotyping R	GTGACATAAATTAGGACAGACAGACG	
**FUL1 -F**	GTTTTGCCACAACAACTGGACTC	RT-qPCR
**FUL1 -R**	CTTGCTGCTGTGAAGAACTACC	
**FUL2 -F**	CATGAGATCTCTGTGCTTTGCG	
**FUL2 -R**	ATCCTTTCCATGCAAGAGTCAGT	
MBP10-F	GAATTTCGGGGTTTAGAGAAACAGC	
MBP10-R	GCTGGGAAATGGACTCGTGC	
MBP20-F	CACATTCTCACCACCAACTTCCTAA	
MBP20-R	AGTGATGAGCCTGACCCGAT	
AP2a-F	ATGGGATTGTGGGAAACAAG	
AP2a-R	CTGCTGCATGTGCTGTATCA	
AP2b-F	ACCCAAGCAACCTAGTCCAC	
AP2b-R	CCGGAGAATGTAGGTGCGTT	
AP2c-F	TGGATATTGATTGGCAGCGC	
AP2c-R	TGGAGAATGCAAGTGCGTCT	
CAC-F	CCTCCGTTGTGATGTAACTGG	
CAC-R	ATTGGTGGAAAGTAACATCATCG	
